# Increase of Universality in Human Brain during Mental Imagery from Visual Perception

**DOI:** 10.1371/journal.pone.0004121

**Published:** 2009-01-05

**Authors:** Joydeep Bhattacharya

**Affiliations:** 1 Department of Psychology, Goldsmiths College, University of London, London, United Kingdom; 2 Commission for Scientific Visualization, Austrian Academy of Sciences, Vienna, Austria; University of East Piedmont, Italy

## Abstract

**Background:**

Different complex systems behave in a similar way near their critical points of phase transitions which leads to an emergence of a universal scaling behaviour. Universality indirectly implies a long-range correlation between constituent subsystems. As the distributed correlated processing is a hallmark of higher complex cognition, I investigated a measure of universality in human brain during perception and mental imagery of complex real-life visual object like visual art.

**Methodology/Principal Findings:**

A new method was presented to estimate the strength of hidden universal structure in a multivariate data set. In this study, I investigated this method in the electrical activities (electroencephalogram signals) of human brain during complex cognition. Two broad groups - artists and non-artists - were studied during the encoding (perception) and retrieval (mental imagery) phases of actual paintings. Universal structure was found to be stronger in visual imagery than in visual perception, and this difference was stronger in artists than in non-artists. Further, this effect was found to be largest in the theta band oscillations and over the prefrontal regions bilaterally.

**Conclusions/Significance:**

Phase transition like dynamics was observed in the electrical activities of human brain during complex cognitive processing, and closeness to phase transition was higher in mental imagery than in real perception. Further, the effect of long-term training on the universal scaling was also demonstrated.

## Introduction

It is an accepted notion that human brain is one of the most complex systems. The brain is complex at all organization levels spanning from the morphology and activity patterns of the individual unit (i.e. single neuron) to the formation and dynamics of neuronal assemblies and finally to the circuitry and ensemble activity of large-scale networks where each node represents the collective dynamics of millions of neuronal assemblies. The involvement of large-scale and distributed cortical networks in higher complex cognition is supported by many studies using diverse imaging modalities. However, it is further proposed that co-activation of these multitude of brain areas are most likely to be associated with functional co-operation between these areas. In essence, brain regions do not act in isolation, rather they display large-scale coherent patterns of activity in both space and time (See, for reviews [1], [2], [3], [4].

**Figure 1:**
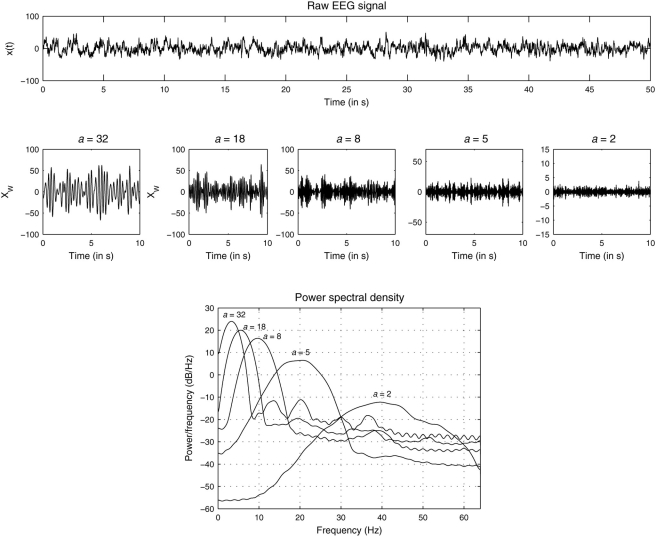
EEG and its (real) wavelet transformed components. *Upper panel*: Segment of electroencephalogram recorded at location Pz from a participant (Vp.483) while imagining an object of visual art shown earlier. *Middle*: Wavelet transform *W_x_*(*a*,*t*) of the signal shown above at five different scales *a* = 32, 18, 10, 5, and 2, respectively. Lower: Power-spectral densities of the wavelet transformed signals.

Even by a cursory look at the noninvasively obtained large scale electrical brain responses (electroencephalogram, EEG, Fig. 1), one could notice an intricate mixture of order (the presence of strong oscillating components) and disorder (the time-varying nature of the amplitude and frequency components of the oscillations). It is known that the oscillatory, yet transient, dynamics of neuronal assemblies emerges from the dense interaction between excitatory and inhibitory sets of neurons, and when modeled, they could produce chaotic oscillations [5], and the flexible switching between multiple chaotic attractors was earlier demonstrated in the olfactory bulb of rabbits [6]. Friston [7] has suggested that brain dynamics could be characterized by a series of flexible neuronal transients where transients represent an essential metric of interaction between neuronal assemblies. In a similar line, Kelso et al [8] showed in a now classic experiment involving bimanual coordination that a phase transition like phenomenon is observed in human brain, suggesting brain as a self-organizing system which operates close to critical points of instability, thereby allowing appropriate flexibility in switching between different dynamical states; this is now known as metastability [2], [7].

Two important properties of complex systems close to their critical points are [9], [10]:

- *scaling* - correlation decays as a power-law 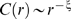 where ξ is the correlation length of the entire system, and the system becomes scale-invariant;
- *universality* - different complex systems have similar critical exponents forming a universal class.

This latter property stems from the fact that a system near its critical point is not very sensitive to the nature of the detailed properties of its components subsystems or to the details of the microscopic interactions, instead it depends on the more fundamental characteristics (i.e. symmetry, dimension, path of order propagation) of the system [11].

The universal behaviour and scale invariance properties seem to be present in numerous real-life systems including natural [12], [13], [14], biological [15], [16], [17], [18], sociological [19] and even political [20] ones, most of which approximately belong to the category of a complex system involving large numbers of interacting subsystems that display the phenomenon of self-organization [21].

In this study, I investigated these two features of criticality in the electrical responses of human brain during higher complex cognition. Towards this, I presented a new approach based on the cumulative variation amplitude analysis [22] to estimate the strength of the universal scaling structure in the time series of multivariate EEG signals. Particularly, I put special emphasis on the comparative analysis of universality during encoding phase (visual perception) and retrieval phase (mental imagery).

## Results

Multivariate EEG signals were recorded from two broad groups – professional artists and non-artists – at three different conditions: (i) visual perception (looking at a painting), (ii) mental imagery (mentally imagining the painting shown before), and (iii) rest. The duration of each condition was not less than 2 min, and after artefact reduction and removal, I analyzed the first 50 sec of spontaneous EEG signals recorded from 19 scalp locations. Fig. 1 shows an EEG signal recorded at scalp location Pz (midline parietal electrode) from an artist (Vp.483) while she was mentally imagining the painting by Holbein which was shown earlier (see Materials & Methods). The wavelet transformed signal with different scales *a* = 32, 18, 10, 5, 2 are shown afterwards. The dominant frequency is not identical to the concept of wavelet scale but they can be closely related: higher scale value is associated with lower frequency and this fact is evident in the power spectra of the wavelet transformed signals. The center frequencies of the wavelet transformed signals for these scale values roughly correspond to the center frequencies of the standard five frequency bands (range), namely, delta (1–4 Hz), theta (4–8 Hz), alpha (8–12 Hz), beta (12–30 Hz) and gamma (>30 Hz). Although the individual power-spectrum of the wavelet transformed signal for a single scale did contain, in addition to a peak at the center frequency, components from neighbouring frequency bands, one could still, for the ease of terminology, associate the wavelet transformed signal predominantly with one of the standard frequency bands. For example, *W_x_*(*a* = 18,*t*) represents theta band oscillations, whereas *W_x_*(*a* = 2,*t*) represents gamma band oscillations. As discussed earlier, the (real) wavelet transform reveals very local properties of the signal by emphasizing the extrema or discontinuities of the oscillations, so the wavelet transformed signal captures the intrinsic local properties of the dynamics masked by nonstationarity, which cannot be revealed by standard stationary digital filtering technique which is globally applied to the signal. For each EEG electrode and for each wavelet scale, I calculated the instantaneous amplitudes *m*(*t*) of the wavelet transformed signal as described in the Eq. (3) and estimated its pdf (*P*(*y*)). Next I grouped pdfs either across different scalp locations within one individual or across individuals at each scalp location. The first one explored the universality across brain regions, and the latter one explored the universality across individuals.

### Task Related Differences

#### Universality Across Brain Regions

**Figure 2:**
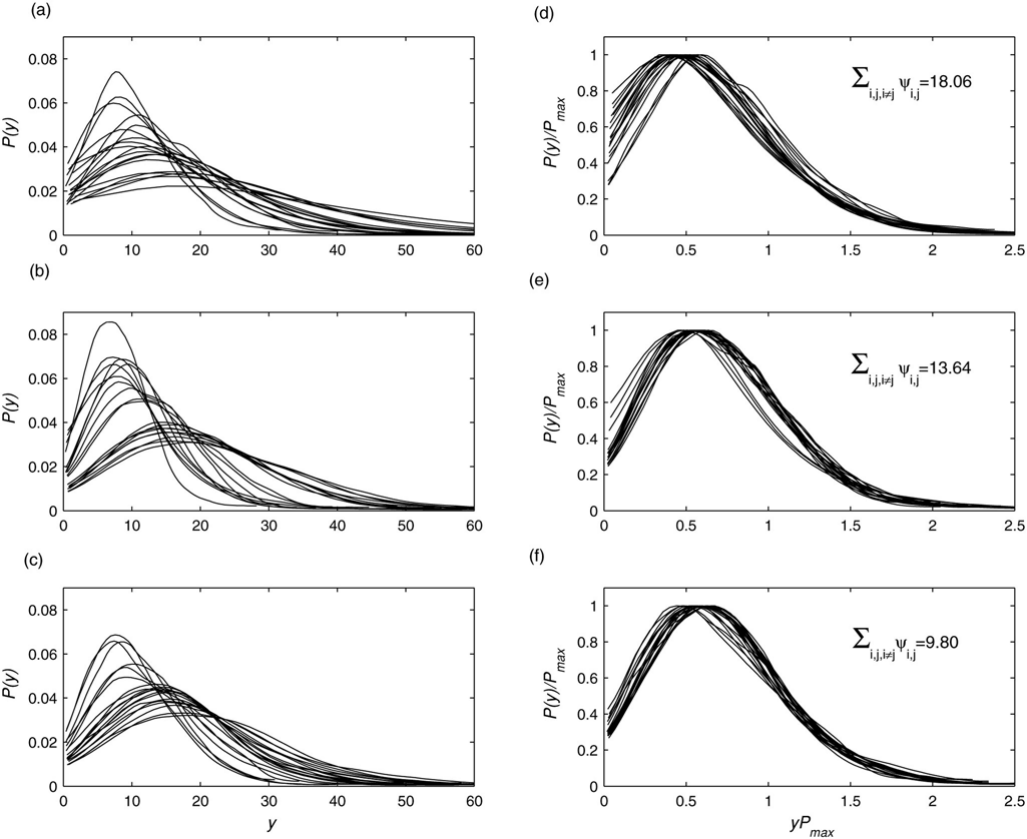
Universality across brain regions for all participants. Empiriical probability density functions *P*(*y*) of the envelope of wavelet transformed coefficients at scale *a* = 2 for multivariate EEG signals recorded from 19 scalp locations from a participant (Vp.483) during (a) resting condition, (b) perception of a visual art object, and (c) mental imagery of the same art object. All pdfs were normalized to unit area. (d–f) Same pdfs are in (a–c) but after rescaling: *P*(*y*) by *P*_max_ and *y* by 1/*P*_max_ to preserve the normalization to unit area. The values in inset indicate the degree of data collapsing as measured by the KL divergence measure (see the text for details). Lower divergence or higher data collapse was found during mental imagery.

Fig. 2(a)–(c) shows a set of pdfs obtained from 19 EEG signals recorded from a non-artist during resting condition, looking at a painting, and mentally imagining the painting shown before, respectively. The wavelet scale was *a* = 18. Inspection of the pdfs reveals marked differences among different brain regions for each condition. These discrepancies are not utterly surprising given the underlying functionally segregative behavior of individual brain regions. To test the hypothesis that there is a hidden, possibly universal structure to these time series generated by distributed brain regions, I rescaled each pdf and computed the Kullback-Leibler (KL) divergence measure for the set of rescaled pdfs (see Methods for details). If the rescaled pdfs collapse, i.e. they are scale invariant, KL measure for the set will be minimal. Here, I found strongest scale invariance or universal structure during mental imagery condition followed by visual perception and resting condition.

**Figure 3:**
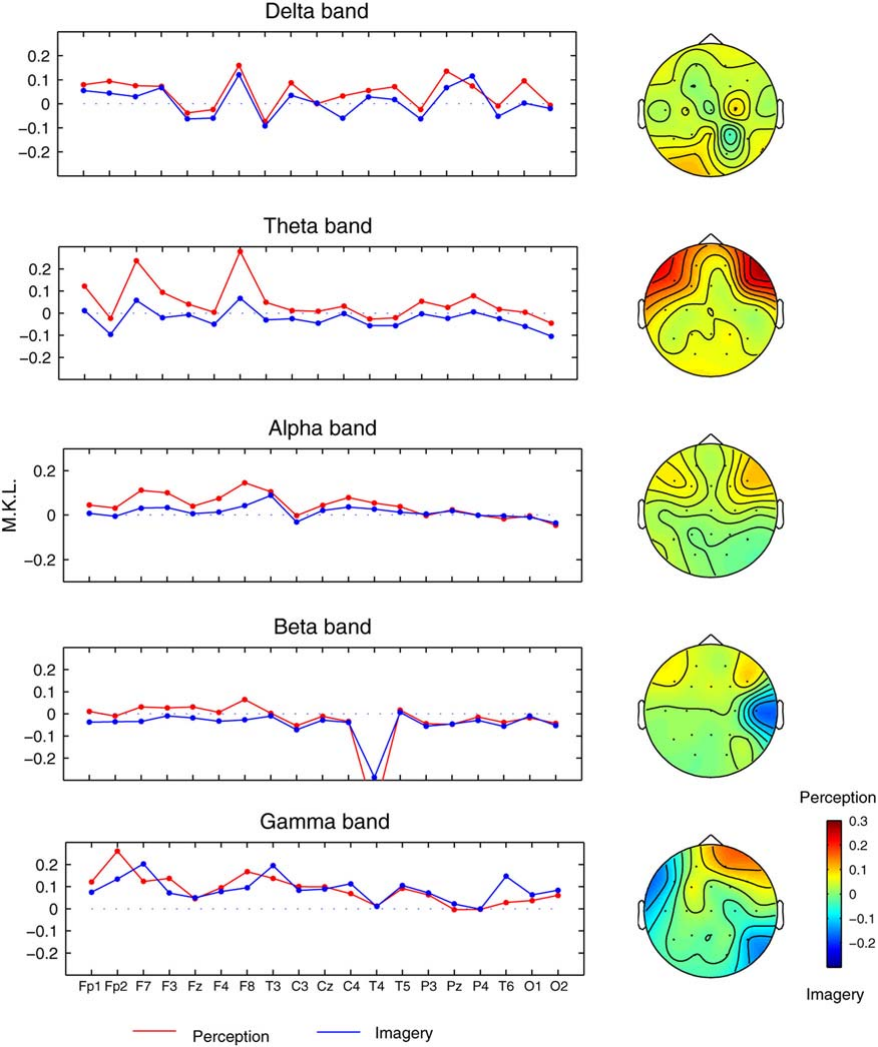
Universality across brain regions at different frequency bands for all participants. Mean Kullback Leibler (MKL) divergence measure for five different scales (*a* = 32, 18, 10, 5, and 2) used in the wavelet transform which roughly correspond to five standard frequency bands: delta (<4 Hz), theta (5–8 Hz), alpha (9–12 Hz), beta (13–30 Hz), and gamma (>30 Hz). Results were pooled across groups, participants, electrode pairs.

The entire analyses were repeated with the chosen five wavelet scales, and Fig. 3 shows the profiles of the Mean-Kullback-Leibler (MKL) divergences averaged over all participants and over all possible combinations of each electrode region for both visual perception and mental imagery. The profiles were plotted after subtracting the MKL values for resting condition. Scalp topographies of the differential (perception minus imagery) MKL are also shown in Fig. 3. Following noteworthy points are found. (i) The degree of universality was overall higher (i.e. MKL values are lower) in mental imagery than in visual perception condition (Wilcoxon signed rank test, *p* = 0.038). (ii) Among frequency bands, this effect was mostly pronounced in low frequency theta band (Wilcoxon, *p* = 0.013, mean difference (perception – imagery) in MKL, Δ_MKL_ = 0.07, which is 11% if expressed in percentage changes with respect to perception condition), followed but less significantly in alpha (Δ_MKL_ = 0.03, 5.1%) and beta bands Δ_MKL_ = 0.01, 4.0%). (iii) Among scalp locations, frontal brain regions, bilaterally, were associated with least universality during visual perception. Since the differences between visual perception and mental imagery were most pronounced in the theta band (*a* = 18), I focussed the subsequent analysis only at this frequency band.

### Group Related Differences

#### Universality Across Brain Regions

**Figure 4:**
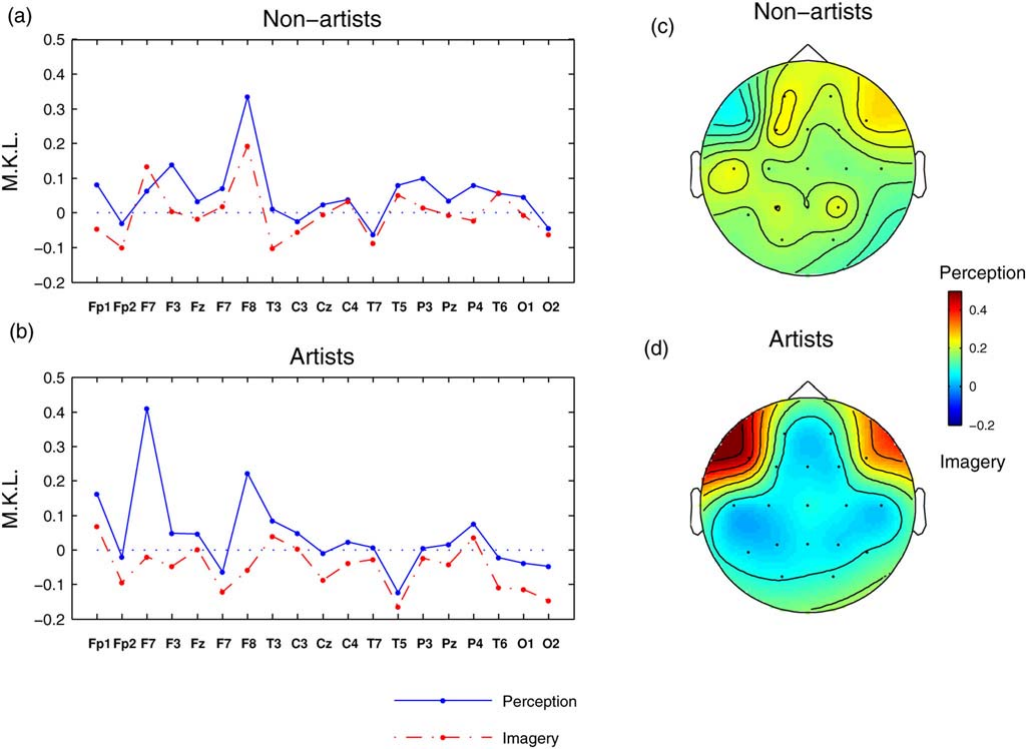
Universality in theta band for artists and non-artists. MKL divergence measure in theta band oscillations for (a) non-artists and (b) artists, respectively. The results were averaged across participants within group, electrode pairs. Note the overall decrease of divergence, i.e. increase of universality for mental imagery condition as compared to visual perception condition. (c–d) Topographical profiles for (a–b). Strongest divergence was observed in frontal regions in both hemispheres for the artists.

The earlier results emphasized the differences between perception and imagery after pooling the data from all participants, where as the group related effects are shown in Fig. 4. It is evident that the scale invariance properties during mental imagery w.r.to visual perception was more pronounced in artists than in non-artists. Further, artists also showed higher (Wilcoxon *p*<0.0039) universality in mental imagery as compared to resting condition (i.e. the MKL profile for artists was mostly negative where as it was more positive for non-artists). On topographic scales, bilateral frontal regions (F7, F8) in artists indicate reduced (paired Wilcoxon *p*<0.002) universal structure during visual perception from mental imagery. In non-artists, the least degree of universality was found in right frontal region (F8, Wilcoxon *p*<0.037 for perception vs rest and *p*<0.013 for imagery vs rest).

#### Universality Across Participants

**Figure 5:**
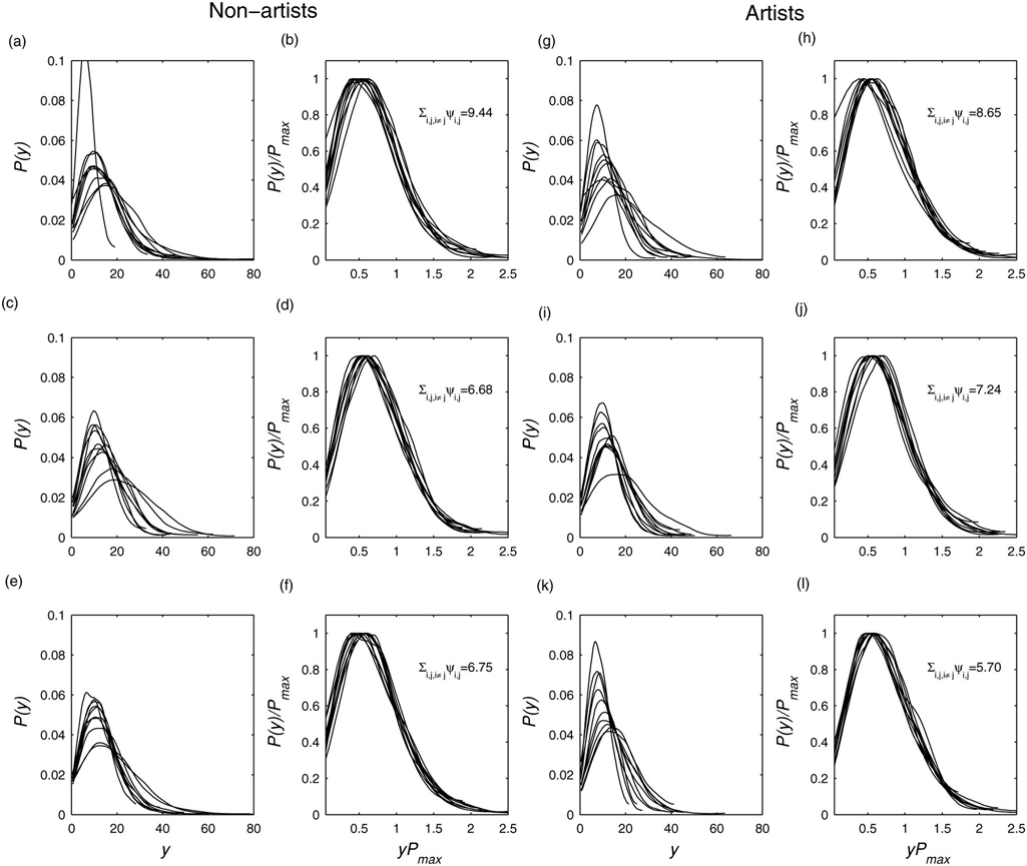
Universality across participants. (a),(c),(e) Empirical probability density functions *P*(*y*) of the instantaneous amplitude of wavelet transformed coefficients at scale *a* = 18 for electrode O2 for a group of non-artists during resting condition, visual perception, and mental imagery, respectively. (b),(d), (f) Same pdfs as earlier but after suitable rescaling: *P*(*y*) by *P*_max_, and *y* by 1/*P*_max_ to preserve the normalization to unit area. The values in inset indicate the degree of data collapsing as measured by the summed KL divergence measure (see the Materials and Methods for details). (g)–(l) The same as in (a)–(f) but for the group of professional artists. Stronger data collapsing were found during mental imagery.

**Figure 6:**
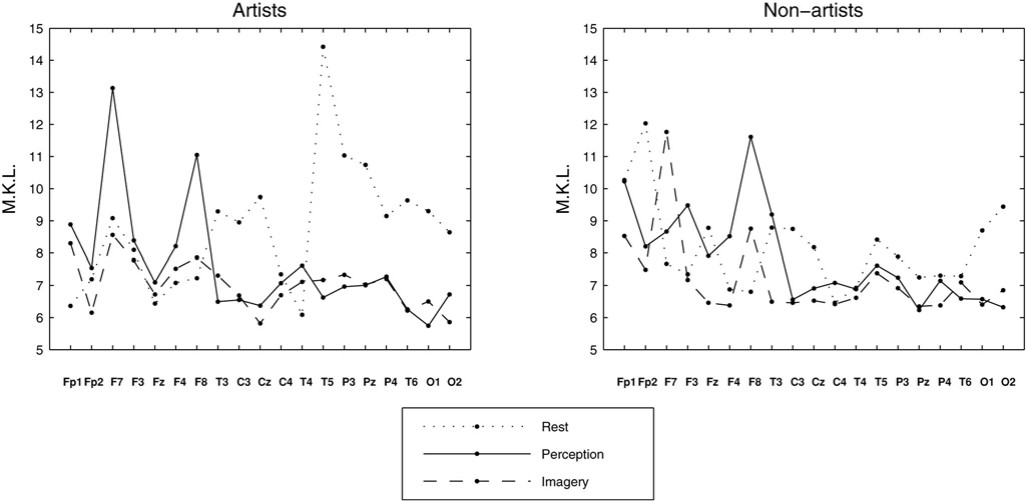
Universality of individual brain regions across participants. Universality across participants as measured by MKL divergence measure for the two groups, (a) artists and (b) non-artists, during three states, rest (dotted line), visual perception (solid), and mental imagery (dash-dot), respectively.

Next I grouped the pdfs of each individual electrode region across participants within each group; the results for electrode region O2 are shown in Fig. 5. For this posterior brain region, data collapsing behaviour was significantly enhanced for both perception and imagery conditions from rest. Fig. 6 shows the degree of universality of individual brain region across participants but within each group. For artists, task-related increases (perception or imagery from rest) in the degree of data collapsing were found in primarily posterior electrode regions excluding T4. Non-artists also showed similar effect, but to a much lesser extent, of weaker data collapsing at rest in the posterior electrode regions. For artists, frontal regions bilaterally (F7 and F8) showed least data collapsing during perception, where as this effect was mostly right accentuated for non-artists.

### Surrogate Analysis

**Figure 7:**
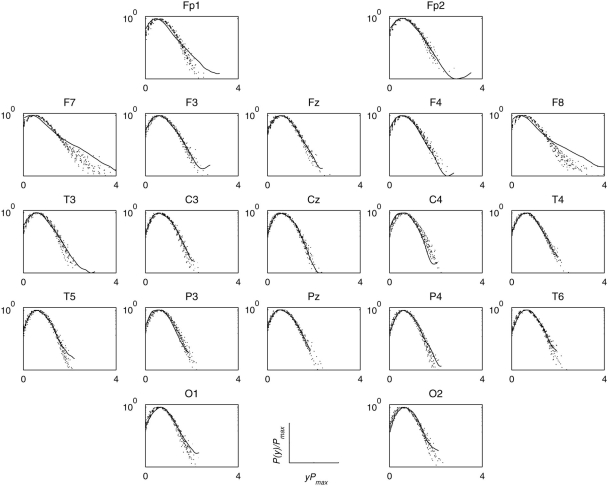
Surrogate analysis during visual perception. Rescaled pdfs (in the semi-log scale) of the envelope of wavelet transformed coefficients at scale *a* = 18 for multivariate EEG signals recorded from 19 scalp locations from a participant (Vp.483) during looking at a painting and for the set ( = 19) of surrogates. The originals were shown in solid line and the surrogates in dotted lines. The long tail of the original pdfs in the frontolateral electrode regions (F7, F8) is conspicuously absent in the pdfs of their surrogates, indicating non-random phase correlations. Note other electrode regions produced.

**Figure 8:**
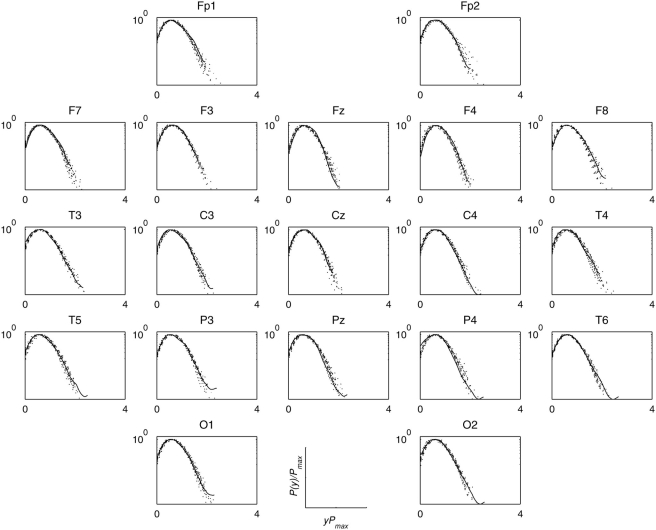
Surrogate analysis during mental imagery. Same as in Fig. 7 but during mental imagery. Note that the pdfs for frontal electrode regions being indistinguishable from those of surrogates and of other electrode regions, which is in sharp contrast with visual perception (Fig. 7).

As a further statistical control, I generated 19 sets of surrogate signals for each set of 19 EEG signals obtained from each individual and for each condition, and compared the data collapse (at a scale *a* = 18) behaviour of surrogates to the original data. Fig. 7 shows one such comparison for an artist (Vp.483) while looking at a painting; the same comparison is displayed in Fig. 8 for mentally imagining the same painting. If a pdf of any electrode region is found to be significantly different from the set of pdfs of surrogates, one can reasonably infer that the phase correlations in this electrode region to be non-random and different from the phases of other electrode regions. Interestingly, the frontal electrode regions, bilaterally (F7, F8, Fp1, Fp2), showed long tailed distributions which could not be reproduced by the surrogate signals, thus suggesting these electrode regions most likely possess not only non-random but also different phase structure from other electrode regions. However, these effects were not found during mental imagery (Fig. 8) since their pdfs were almost indistinguishable from those surrogate pdfs and also from pdfs of other electrode regions. These effects were found to be remarkably consistent across artists. These altogether suggest that, as compared to other electrode regions, frontal electrode regions in artists possess distinctly different phase correlation during visual perception but similar phase correlations during mental imagery.

## Discussion

The overall similarities in the topological profile of mean Kullback-Leibler measure across several frequency bands during visual perception and mental imagery support the hypothesis that the diverse brain regions active during sensory-induced perceptions are reactivated during retrieval of such information [23], [24], [25]. However, I also found that this similarity was less in artists at the frontal regions for theta band oscillations where mental imagery was found to induce stronger universal structure than visual perception. Or conversely, the degree of universality at frontal regions was minimal across all brain regions in artists during visual perception. It could be explained as follows. Visual perception of art broadly consists of three stages [25]: (i) extraction of basic visual features, (ii) organization of these features into coherent and fundamental forms, followed by (iii) addition of meaning onto these forms through associations stored in long-term memory. This last stage can be termed as top-down processing where the brain adds the information to raw visual impressions giving a richness of meaning well beyond the sensory stimuli [26]. Prefrontal cortex plays a crucial role by providing this top-down control [27], and it is reasonable to assume that the involvement of prefrontal cortex during perception of visual art would vary substantially across artists due to their expertise and training in visual art. However, the top-down control operation was significantly reduced during mental imagery of the painting shown before, thus leading to a substantial increase of universality in frontal regions. Further, low frequency oscillation in theta band plays a prominent role in mediating access to stored representation in long term memory [28], and moreover, an increase in theta band oscillation in frontal regions was found during concentrated mental activity requiring higher memory load [29]. During imagery, the extent of retrieved visual-art patterns from long-term memory was assumed to be much higher in artists than in non-artists which possibly led to an increase of universality.

Several studies have earlier shown that ongoing spontaneous brain activity exhibits scale-free behaviour at resting condition [30], [31], [32], where scale-free dynamics is primarily characterized by a power-law scaling behaviour of amplitude fluctuations in raw signal or in specific frequency bands. Recent evidence also indicates that scale-free structure can be altered by external stimulus, such as nerve stimulation [33], performance feedback [34], music [35], imaginary and visual motor-tracking [36]. Noteworthy to mention that all these studies report a stimulus related modulation, but not a disruption, of the scaling activity. The present study extends this finding by reporting that not only the scale-free properties but also the underlying universality could be modulated by complex cognitive tasks and by task-specific expertise.

But what can one conclude by finding such universal structure in large scale brain responses? Before answering this question, let me mention a few key details about universality. Universality, as discussed here, refers to a phenomenon whereby different systems exhibit very similar characteristic or critical exponents which determine the correlation and scaling functions [9], [37]. Since critical exponents offer a complete description of the dynamics of a system near a continuous phase transition including the emergence of a long-range interaction out of paradoxical competition between exponentially decaying correlation function and exponentially increasing number of connecting paths. Two systems with similar scaling functions and critical exponents belong to the same universality class. Thus, if the critical properties of one system could be known, it would theoretically be possible to predict the critical properties of the other system belonging to the same universality class [38]. The finding that the artists group showed a stronger universality across brain regions during mental imagery offer a surprising conclusion: despite the possibilities of wide individual variations across many hidden degrees of freedom in the group of artists, during mental imagery there exists a remarkable consistency in dynamical scaling and correlation characteristics across different brain regions distributed over the scalp.

Finally, I would like to leave two cautionary remarks. First, Kadanoff showed [10] that scaling and universality of critical exponents are primarily a consequence of the scale invariance of physical systems near critical points, but the converse is not essentially true; in other words, by observing some sort of universality, one cannot prove the closeness to criticality and the presence of a scale invariance. Second, the adopted experimental paradigm involved a very highly abstract task of complex cognition lasting for minutes, so one has to be careful with interpretations before “overstretching” the findings. The very nature, i.e. ecologically valid and naturally appropriate, of the task/paradigm, and the nature of the recorded signals (i.e. from large scale EEG signals, it is almost impossible to prove the theoretical concept of universality as produced by two trajectories of two neuronal populations, the local manifolds of which have similar Jacobians close to their critical points though a novel way was offered to find the closeness to the universality) impose the constraints, yet a more pragmatic approach would be to look for the consistency and systematic differences across tasks and groups.

In summary, a new method has been discussed to find the hidden universal structure in a multivariate data set. The paper also presents evidence that conceptual framework provided by the theory of statistical mechanics to characterize complex systems poised at criticality by twin pillars of scaling and universality may be useful in providing new insights into the analysis of brain electrical responses recorded under complex cognitive task paradigm.

## Materials and Methods

### Participants and Stimuli

Forty three female participants were divided into two groups: (i) artists (*n* = 19, mean age 38.4 yrs) with M.A. degree in Fine Arts, and (ii) non-artists (*n* = 24, mean age 36.6 yrs) without any training or prominent interest in visual art. Three conditions were considered: (1) *visual perception*: looking at slides of four paintings characterizing four different periods in the history of the Fine arts (Bean-festival by Jordeans, a charcoal-etching by Rembrandt, a portrait by Holbein, and an abstract figure by Kandinksy), (2) *mental imagery*: mental imagination of these paintings shown before, and (3) *rest*: resting with eyes opened. At the end of each condition, the participants read a newspaper article for distraction. Each of the condition lasted for at least 2 min and the orders of the tasks as well as the orders of the paintings were randomized. All participants gave informed written consent and the study was formally approved by the local ethics committee of the Brain Research Institute, University of Vienna, Austria.

### Data recording

Multivariate (19 channels) EEG signals were recorded by 19 gold-cup electrodes (Fig. S1) which were equally distributed over the scalp according to the standard 10–20 electrode placement system [39] with respect to the forehead as ground. EEG signals were amplified by Nihon-Kohden amplifier. The signals were later algebraically re-referenced by the average signals of two ear-lobes. The electrode impedance was kept below 8 kOhm, the sampling frequency was 128 Hz and the A/D conversion was 12 bit. Custom-made independent component analysis based software was utilized offline to remove the eye-blink related components and other artefacts.

### Data processing

The data processing was composed of four steps as follows.

#### 1. Wavelet analysis

The wavelet analysis is analogous in nature to the Fourier analysis by which a signal is decomposed in to a set of finite basis functions. The primary advantage of wavelet is the local property of the chosen wavelet basis function which may be appropriate to detect transient dynamics in the signal, where as such transient is often obscured by the fixed trigonometric basis function with infinite support used in the Fourier analysis. Let *x*(*t*) be the signal and Ψ be the mother wavelet. The wavelet coefficients *W_x_*(*a*,*t*) are produced through convolution of the scaled mother wavelet function with the analyzed signal as follows:
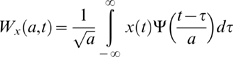
(1)where *a* is the scale of the wavelet which is inversely related to frequency, and *t* is the local time origin of the analyzed wavelet.

The nature of oscillation with the continuous wavelet spectrum will depend crucially on the nature, complex or real, of the mother wavelet. The complex wavelet usually produces a constant power across the entire time duration of the oscillation, whereas a real wavelet produces power mostly at those times where the oscillation is at an extreme or where a sharp discontinuity occurs.

In this study, I used real Morlet wavelet, which has [−4,4] as effective support, and is defined as an exponentially decaying sinusoidal signal: 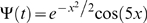. There are numerous other wavelets that could also be adopted [40]; however, the Morlet wavelet is particularly suitable for oscillatory signals generated by dynamical systems. Typically, complex Morlet wavelet has been routinely used in estimating time-varying spectral content in brain oscillations [41], [42], whereas real wavelets are better suited to detect sharp signal transitions. Matlab® function cwt was used to compute the continuous wavelet transformed coefficients.

#### 2. Analytic signal formation

The second step of the analysis was to extract the instantaneous variation amplitude of the wavelet transformed signal by means of an analytic signal approach [43]. For any wavelet transformed signal *W*_x_(*a*,*t*) at scale *a*, an analytic signal, *z*(*t*), is defined as
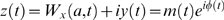
(2)in which
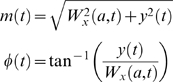
(3)Theoretically, there are infinitely main ways of defining the imaginary part *y*(*t*), but the Hilbert transform provides a unique way of defining the imaginary part so that the result would become a complex analytic function. Further, Hilbert transform (H.T.) is particularly attractive because it does not require any information concerning the centre frequency of the signal. For the sequence *W_x_*(*a*,*t*), Hilbert transform is calculated as
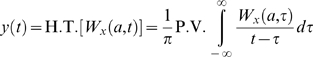
(4)where P.V. means that the integral is taken in the sense of the Cauchy Principal Value. From the above equation, the Hilbert transform *y*(*t*) can be considered as the convolution of the concerned time series with 1/π*t*, and does not produce a change of domain unlike Fourier transform which changes from time domain to frequency domain representation. Hilbert transform can be realized by a ideal filter whose amplitude response is unity and phase response is a constant π/2 lag at all frequencies [44]. Matlab® function hilbert was used to calculate the analytic signal.

The concept of analytic signal may also be better understood by comparing the role of phasors in simplifying manipulations of current and voltages [45]. A rotating phasor is defined by
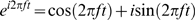
(5)Comparing Eq.(2) to Eq.(5), it is evident that the given real signal *W_x_*(*a*,*t*) and its Hilbert transformed version *y*(*t*) play analogous role to the real part and the imaginary part of an unit phasor. This fact can be explained as follows. Let us consider a point undergoing uniform circular motion about the centre. The projection of the motion of which onto axes at right angles to one another yields the pair of sine and cosine waves, respectively. If the time axes are combined, and the sine and cosine waves are included and their instantaneous values are projected into the three dimensional space, then the motion of the resulting point traces out the trip of the vector originating at the time axis. The resulting vector is termed as the analytic signal, and its length is the amplitude of the analytic signal. Ideally the analytic procedure put the low frequency content in to the amplitude *a*(*t*) and the high frequency component in to the phase φ(*t*) [43].

Here I investigated only the instantaneous amplitude component and its empirical probability density function (pdf) was studied next.

#### 3. Empirical Probability Density Function (PDF) analysis

For each electrode, task and participant, I computed the pdf *P*(*y*) of instantaneous amplitudes of the wavelet transformed signal and normalized it to unit variance. For individual task condition (rest/perception/imagery), these individual pdf's were pooled together according to scalp locations or participants within each group (artist/non-artist). To test the hypothesis that there is a hidden, possibly universal, structure to these different pdf's, the pdf's were rescaled as follows: *P*(*y*) by *P*_max_ and *y* by 1/*P*_max_ to preserve the normalization to unit area [22]. If there exists, indeed, a universal structure among all these pdf's, the rescaled pdf's would collapse into a single pdf and the entire pool of pdf's can be described a single scaling parameter.

Such collapsing of density functions is reminiscent of a wide-class of well-studied physical and natural systems with universal scaling properties [9]. This stems from the fact that generalized homogenous functions display this property of scale invariance and data collapsing, and such functions were investigated in the context of formalism to treat thermodynamic functions, static correlation function, dynamic correlation function and universality near the critical point [9].

Lets mention briefly the key features of generalized homogenous function [46]. A function *f*(*x*,*y*,*z*, …) of any number of variables is called homogeneous of degree *m* in these variables if multiplication of each of the variables *x*, *y*, *z*, …by a positive scalar λ results in multiplication of the function by λ*^m^*, i.e.
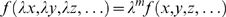
(6)where the parameter *m* is generally called the degree of homogeneity. Now this function will be called a generalized homogeneous function if one finds a set of numbers *a*, *b*, *c*, … not all zero, such that
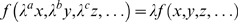
(7)


However, it needs to be stressed here that one would rarely obtain a strict data collapsing behaviour of pdf's when dealing with real-life ongoing EEG signals, and even more so when the underlying task was as complex as visual perception and mental imagery of complex object like a painting. Therefore, it was more appropriate to perform a comparative study, i.e. whether data collapsing was more (or less) in visual perception than in mental imagery. In order to quantify the degree of data collapsing, I used Kullback-Leibler (KL) divergence measure [47] which is also known as relative entropy or cross-entropy [48]. KL between two pdfs *P*(*x*) and *Q*(*x*) over the same alphabet Λ*_X_* is
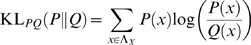
(8)KL is always non-negative and is zero iff *P* = *Q*. So the degree of similarities between two pdfs is inversely related to the value of KL. Therefore, if I computed the KL for all pair-wise pdf's within each pool, the averaged or mean-Kullback-Leibler (MKL) measure approximately quantifies the degrees of universality or scale invariance of that pool. The lower the values of MKL, the higher the degrees of universality and scale invariance.

#### 4. Surrogate Analysis

The final analysis was based on the method of surrogate data analysis [49], which is an application of the popular statistical bootstrapping technique [50]. The surrogate series are generated from the original EEG signals on the basis of a certain null hypothesis. A most basic null hypothesis is that EEGs are completely random, and a more advanced null hypothesis is that EEG signals are generated by filtered Gaussian linear stochastic processes, which may be observed through a static nonlinear but invertible transformation function. Here, surrogates possess all the linear structure (mean, variance, power spectrum and circular autocorrelation) but are devoid of any phase correlations present in the original signal. Since multiple EEG signals were measured simultaneously, it would be important to incorporate also the phase correlation properties between multiple EEG signals [51]. Here the set of surrogates not only possess the earlier mentioned linear structure of individual EEG signals but also their cross spectral information. In this study, I generated 19 surrogates for each EEG signal and their pdfs of the instantaneous amplitude of the wavelet transform using the same scale (*a*) were compared to the corresponding pdf of the original signal. If the original pdf is indistinguishable from the set of surrogates, one can accept the underlying null hypothesis of linear Gaussian process.

## Supporting Information

Figure S1 Electrode locations. Topographical locations of the 19 electrodes and their designations according to the International 10–20 systems.
(0.01 MB EPS)

## References

[1] Varela F, Lachaux JP, Rodriguez E, Martinerie J (2001). The brainweb: Phase synchronization and large-scale integration.. Nature Reviews Neuroscience.

[2] Bressler SL, Kelso JAS (2001). Cortical coordination dynamics and cognition.. Trends in Cognitive Sciences.

[3] Schnitzler A, Gross J (2005). Normal and pathological oscillatory communication in the brain.. Nat Rev Neurosci.

[4] Pereda E, Quian Quiroga R, Bhattacharya J (2005). Nonlinear multivariate analysis of neurophysiological signals.. Prog Neurobiol.

[5] van Vreeswijk C, Sompolinsky H (1996). Chaos in neuronal networks with balanced excitatory and inhibitory activity.. Science.

[6] Freeman WJ, Skarda CA (1985). Spatial EEG patterns, non-linear dynamics and perception: the neo-Sherringtonian view.. Brain Res.

[7] Friston KJ (1997). Transients, metastability, and neuronal dynamics.. Neuroimage.

[8] Kelso JAS, Bressler SL, Buchanan S, Deguzman GC, Ding M (1992). A Phase-Transition in Human Brain and Behavior.. Physics Letters A.

[9] Stanley HE (1971). Introductions to Phase Transitions and Critical Phenomena.

[10] Kadanoff LP (1966). Scaling laws of Ising models near Tc.. Physics.

[11] Wilson KG (1979). Problems in physics with many scales of length.. Scientific American.

[12] Li W, Csathy GA, Tsui DC, Pfeiffer LN, West KW (2005). Scaling and universality of integer quantum Hall plateau-to-plateau transitions.. Phys Rev Lett.

[13] Corral A (2004). Long-term clustering, scaling, and universality in the temporal occurrence of earthquakes.. Phys Rev Lett.

[14] Murphy D, Genio E, Ahlers G, Liu F, Liu Y (2003). Finite-size scaling and universality of the thermal resistivity of liquid 4He near Tlambda.. Phys Rev Lett.

[15] Banavar JR, Damuth J, Maritan A, Rinaldo A (2002). Ontogenetic growth: Modelling universality and scaling.. Nature.

[16] Ivanov P, Rosenblum MG, Peng CK, Mietus JE, Havlin S (1998). Scaling and universality in heart rate variability distributions.. Physica A.

[17] Kiyono K, Struzik ZR, Aoyagi N, Sakata S, Hayano J (2004). Critical scale invariance in a healthy human heart rate.. Phys Rev Lett.

[18] Gisiger T (2001). Scale invariance in biology: coincidence or footprint of a universal mechanism?. Biol Rev Camb Philos Soc.

[19] Bettencourt LM, Lobo J, Helbing D, Kuhnert C, West GB (2007). Growth, innovation, scaling, and the pace of life in cities.. Proc Natl Acad Sci U S A.

[20] Fortunato S, Castellano C (2007). Scaling and universality in proportional elections.. Phys Rev Lett.

[21] Bak P (1996). How nature works : the science of self-organized criticality.

[22] Ivanov PC, Rosenblum MG, Peng CK, Mietus J, Havlin S (1996). Scaling behaviour of heartbeat intervals obtained by wavelet-based time-series analysis.. Nature.

[23] Kosslyn SM (1980). Image and Mind.

[24] Kosslyn SM, Ganis G, Thompson WL (2001). Neural foundations of imagery.. Nature Reviews Neuroscience.

[25] Bhattacharya J, Petsche H (2002). Shadows of artistry: cortical synchrony during perception and imagery of visual art.. Cognitive Brain Research.

[26] Espinel CH (1998). Art and neuroscience: how the brain sees Vermeer's Woman Holding a Balance.. Lancet.

[27] Frith C, Dolan RJ (1997). Brain mechanisms associated with top-down processes in perception.. Philosophical Transactions of the Royal Society of London Series B-Biological Sciences.

[28] Doppelmayr M, Klimesch W, Schwaiger J, Stadler W, Rohm D (2000). The time locked theta response reflects interindividual differences in human memory performance.. Neurosci Lett.

[29] Gevins A, Smith ME, McEvoy L, Yu D (1997). High-resolution EEG mapping of cortical activation related to working memory: effects of task difficulty, type of processing, and practice.. Cereb Cortex.

[30] Nikulin VV, Brismar T (2005). Long-range temporal correlations in electroencephalographic oscillations: Relation to topography, frequency band, age and gender.. Neuroscience.

[31] Hwa RC, Ferree TC (2002). Scaling properties of fluctuations in the human electroencephalogram.. Phys Rev E Stat Nonlin Soft Matter Phys.

[32] Linkenkaer-Hansen K, Nikouline VV, Palva JM, Ilmoniemi RJ (2001). Long-range temporal correlations and scaling behavior in human brain oscillations.. J Neurosci.

[33] Linkenkaer-Hansen K, Nikulin VV, Palva JM, Kaila K, Ilmoniemi RJ (2004). Stimulus-induced change in long-range temporal correlations and scaling behaviour of sensorimotor oscillations.. Eur J Neurosci.

[34] Buiatti M, Papo D, Baudonniere PM, van Vreeswijk C (2007). Feedback modulates the temporal scale-free dynamics of brain electrical activity in a hypothesis testing task.. Neuroscience.

[35] Bhattacharya J, Petsche H (2001). Universality in the brain while listening to music.. Proceedings of the Royal Society of London Series B-Biological Sciences.

[36] Popivanov D, Stomonyakov V, Minchev Z, Jivkova S, Dojnov P (2006). Multifractality of decomposed EEG during imaginary and real visual-motor tracking.. Biol Cybern.

[37] Binney J (1992). The Theory of critical phenomena : an introduction to the renormalization group.

[38] Werner G (2007). Metastability, criticality and phase transitions in brain and its models.. Biosystems.

[39] Jasper HH (1958). Report of the committee on methods of clinical examination in electroencephalography.. Electroencephalography and Clinical Neurophysiology.

[40] Mallat SG (1999). A wavelet tour of signal processing.

[41] Tallon-Baudry C, Bertrand O, Delpuech C, Permier J (1997). Oscillatory gamma-band (30–70 Hz) activity induced by a visual search task in humans.. J Neurosci.

[42] Bhattacharya J, Shams L, Shimojo S (2002). Sound-induced illusory flash perception: role of gamma band responses.. Neuroreport.

[43] Cohen L (1995). Time-frequency analysis.

[44] Panter PF (1965). Modulation, noise, and spectral analysis, applied to information tranmission.

[45] Haykin SS (2001). Communication systems.

[46] Gelfand IM, Shilov GE, Graev MI, Vilenkin NIFAF (1964). Generalized functions.

[47] Kullback S (1959). Information theory and statistics.

[48] MacKay DJC (2004). Information theory, inference, and learning algorithms.

[49] Theiler J, Eubank S, Longtin A, Galdrikian B, Farmer JD (1992). Testing for Nonlinearity in Time-Series - the Method of Surrogate Data.. Physica D.

[50] Efron B, Tibshirani R (1993). An introduction to the bootstrap.

[51] Prichard D, Theiler J (1994). Generating Surrogate Data for Time-Series with Several Simultaneously Measured Variables.. Physical Review Letters.

